# Ethnomedicinal and ecological status of plants in Garhwal Himalaya, India

**DOI:** 10.1186/1746-4269-7-32

**Published:** 2011-10-19

**Authors:** Munesh Kumar, Mehraj A Sheikh, Rainer W Bussmann

**Affiliations:** 1 Department of Forestry, HNB Garhwal University, Srinagar Garhwal, Uttarakhand, India; 2 William L. Brown Center, Missouri Botanical Garden, St. Louis, MO 63110, USA

## Abstract

**Background:**

The northern part of India harbours a great diversity of medicinal plants due to its distinct geography and ecological marginal conditions. The traditional medical systems of northern India are part of a time tested culture and honored still by people today. These traditional systems have been curing complex disease for more than 3,000 years. With rapidly growing demand for these medicinal plants, most of the plant populations have been depleted, indicating a lack of ecological knowledge among communities using the plants. Thus, an attempt was made in this study to focus on the ecological status of ethnomedicinal plants, to determine their availability in the growing sites, and to inform the communities about the sustainable exploitation of medicinal plants in the wild.

**Methods:**

The ecological information regarding ethnomedicinal plants was collected in three different climatic regions (tropical, sub-tropical and temperate) for species composition in different forest layers. The ecological information was assessed using the quadrate sampling method. A total of 25 quadrats, 10 × 10 m were laid out at random in order to sample trees and shrubs, and 40 quadrats of 1 × 1 m for herbaceous plants. In each climatic region, three vegetation sites were selected for ecological information; the mean values of density, basal cover, and the importance value index from all sites of each region were used to interpret the final data. Ethnomedicinal uses were collected from informants of adjacent villages. About 10% of inhabitants (older, experienced men and women) were interviewed about their use of medicinal plants. A consensus analysis of medicinal plant use between the different populations was conducted.

**Results:**

Across the different climatic regions a total of 57 species of plants were reported: 14 tree species, 10 shrub species, and 33 herb species. In the tropical and sub-tropical regions, *Acacia catechu *was the dominant tree while *Ougeinia oojeinensis *in the tropical region and *Terminalia belerica *in the sub-tropical region were least dominant reported. In the temperate region, *Quercus leucotrichophora *was the dominant tree and *Pyrus pashia *the least dominant tree. A total of 10 shrubs were recorded in all three regions: *Adhatoda vasica *was common species in the tropical and sub-tropical regions however, *Rhus parviflora *was common species in the sub-tropical and temperate regions. Among the 33 herbs, *Sida cordifolia *was dominant in the tropical and sub-tropical regions, while *Barleria prionitis *the least dominant in tropical and *Phyllanthus amarus *in the sub-tropical region. In temperate region, *Vernonia anthelmintica *was dominant and *Imperata cylindrica *least dominant. The consensus survey indicated that the inhabitants have a high level of agreement regarding the usages of single plant. The index value was high (1.0) for warts, vomiting, carminative, pain, boils and antiseptic uses, and lowest index value (0.33) was found for bronchitis.

**Conclusion:**

The medicinal plants treated various ailments. These included diarrhea, dysentery, bronchitis, menstrual disorders, gonorrhea, pulmonary affections, migraines, leprosy. The ecological studies showed that the tree density and total basal cover increased from the tropical region to sub-tropical and temperate regions. The species composition changed with climatic conditions. Among the localities used for data collection in each climatic region, many had very poor vegetation cover. The herbaceous layer decreased with increasing altitude, which might be an indication that communities at higher elevations were harvesting more herbaceous medicinal plants, due to the lack of basic health care facilities. Therefore, special attention needs to be given to the conservation of medicinal plants in order to ensure their long-term availability to the local inhabitants. Data on the use of individual species of medicinal plants is needed to provide an in-depth assessment of the plants availability in order to design conservation strategies to protect individual species.

## Background

The Indian Himalayan Region (IHR) has long been a source of medicine for the millions of people of this region as well as people living in other parts of India. At present, the pharmaceutical sector in India is making use of 280 medicinal plant species, of which 175 are found in the IHR [1].

The northern part of India harbors a great diversity of medicinal plants because of the majestic Himalayan range. So far, about 8000 species of angiosperms, 44 species of gymnosperms, and 600 species of pteridophytes have been reported in the Indian Himalaya [2]. Of these, 1748 species are used as medicinal plants [3], and the maximum number of species used as medicines has been reported from Uttarakhand [4]. Of these, sixty-two are endemic to the Himalaya.

In India, the native people exploit a variety of herbals for effective treatment of various ailments. The plant parts used, preparation, and administration of drugs vary from place to place [5]. Indigenous knowledge is as old as human civilization, but the term *ethnobotany *was coined by an American botanist, John Harshburger [6], who understood the term to mean the study of the plants used by primitive and aboriginal people. Since time immemorial, plants have been employed by traditional medicine in different parts of the world. According to the World Health Organization (WHO), as many as 80% of the world's people depend on traditional medicine to meet their primary health care needs. There are considerable economic benefits stemming from the development of indigenous medicine and the use of medicinal plants for the treatment of various diseases [7]. Medicinal plants have traditionally occupied an important position in the socio-cultural, spiritual, and health arena of rural and tribal India. India has one of the oldest, richest, and most diverse systems of traditional medicine. The use of plants to cure diseases is an age-old practice. The preparation of locally available medicinal plants remains an important part of health care for humans, especially for people living in rural areas, where people lack access to modern medicine facilities, and are unable to afford synthetic drugs due to its high cost. The forests of India have been the source of invaluable medicinal plants since man became aware of the preventive and curative properties of plants and started using them for human health care.

The old Indian Systems of Medicine (ISM) are among the most ancient medical traditions known, and derive maximum formulations from plants and plant extracts found in the forests. About 400 plants are used in the regular production of Ayurvedic, Unani, Siddha, and tribal medicine. About 75% of these are taken from tropical forests and 25% from temperate forests. Thirty (30) percent of ISM preparations are derived from roots, 14% from bark, 16% from whole plants, 5% from flowers, 10% from fruits, 6% from leaves, 7% from seeds, 3% from wood, 4% from rhizomes, and 6% from stems. Fewer than 20% of the plants used are cultivated [8].

The occurrence of diverse ecosystems along altitudinal gradients form the tropical to the temperate and alpine zones with its associated impressive array of species and genetic diversity make India one of the 12 mega-biodiversity countries of the world. Forest represents one of the dominant components of the vegetation of India and forest floras constitute an invaluable reserve of economically important species, harboring traditional varieties and wild relatives of many crops. The wide range of plant species help to provide for people's needs, including the need for medicines.

The changing situation in the various ecological zones, especially the loss of habitat, habitat fragmentation, and habitat degradation is the major threat to plant diversity of the region. In those areas, where human population density is highest, most of the original habitats have already been destroyed, and many of the important medicinal plant species have been lost. The demand for housing, agriculture, and tourism development is also high. Degradation caused by an increase in human activities related to the growing population, and the lack of serious efforts to counteract them is an important concern. Human destruction of natural habitats, migration of human population, invasive species, the growing demand for natural resources and the lack of adequate training on the subject of biodiversity, all these factors are accelerating the loss of plant species. Along with the disappearance of plants from the area, traditional knowledge is also being lost.

The importance of ethnobiological knowledge for suggesting new paths in scientific research on ecology and conservation monitoring, has received much attention in resource management [9,10]. International agencies such as the World Wildlife Fund (WWF) and UNESCO as part of their people and plants initiative, have also promoted research on ethnobotanical knowledge and the integration of people's perceptions and practices in resource management at the local level [11]. Incorporation into biological and ecological studies of local-use patterns and of the social and institutional background that guides the relationships between people and nature, has led to a greater understanding of the relationship between social and ecological dynamics [12].

In the Himalayan region, which is rich in floral diversity, plants are used by the local inhabitants for their daily needs, even as they exploit the forests for different industrial purposes. The people of the Himalayan region are well aware of the traditional use of medicinal plants, but the ecological distribution of the species in the areas surrounding human habitat tell us the rate of its utilization for sustainable long-term use. Although many studies have been carried out on the ethnomedicinal uses of the plants described from the different parts of India and elsewhere [13-20]. However, there have been few ecological studies of medicinal plants in the Himalayan region in general, and none in Garhwal Himalaya. The present study was conducted to understand the ethnomedicinal and ecological status of plants in the region. The study focused on the following: 1).The use of medicinal plants by local inhabitants for various ailments. 2) The ecological status, presence and availability of medicinal plants around the villages for the villagers needs. 3) The level of exploitation by the local inhabitants and possible sustainable conservation measures.

## Materials and methods

### Details of study area

**Figure 1:**
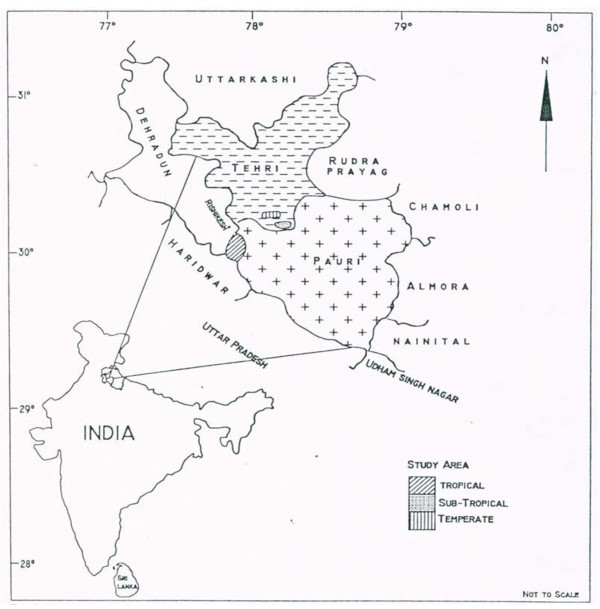
**Location map of the study area**.

Ecological information about medicinal plant species was collected in three different climatic regions of Garhwal Himalaya: tropical, sub-tropical, and temperate regions at an average altitude of 350, 1100, and 2300 m a.m.s.l. (Figure 1), and their medicinal use was documented. The tropical region was primarily flat with a few south west facing hills. The sub-tropical region also faced toward south west. The temperate sites were south east facing. The summer season in the tropical region is very hot and temperatures range between 18-24°C. In sub-tropical region, which is mildly hot in the summer season, temperatures range between 17-23°C, and in temperate region temperatures range between 7-15°C, with some days below freezing in winter (October to February). The tropical region is part of the Pauri Garhwal district in the foothill region of Garhwal Himalaya. The sub-tropical and temperate regions are in Tehri Garhwal district. The total population of the villages was 1140 inhabitants in the tropical, 374 in the sub-tropical and 464 temperate regions respectively. Ten percent of the population (114, 38 and 47) was interviewed. Further details of the regions are given in Table 1.

**Table 1:** Description of study area

Parameter	Tropical	Sub-tropical	Temperate
Location	30° 6' N, 78° 24' E	30° 29' N 78° 24' E	30° 22' N 78° 23' E
Altitude (m.a.s.l.)	350	1100	2300
Aspect	South West	South West	South East
Temperature (mean annual)	24°	17°-23°	7°-15°
Precipitation (mm)	1350	960	1600
Human population	1140	374	464
Total informants	114	38	47
Average family size	6	5	6

### Data collection and analysis

#### Vegetation

Ecological data indicating the species composition in different forest layers were collected from each region. The species composition (Table 2) was assessed with the help of quadrate sampling method. A total of 25, 10 × 10 m quadrats were selected randomly to assess trees and shrubs, and 40, 1 × 1 m quadrats were used for herbaceous plants. The vegetation data were quantitatively analyzed for density, total basal cover (TBC) [21], and the importance value index (IVI) was calculated as the sum of relative frequency, relative density and relative dominance [22]. In each climatic region, three sites were selected, and the mean values of density, basal cover, and importance value index from all sites of each region were used to interpret the final data.

**Table 2:** Density, TBC (total basal cover), IVI (importance value index) of ethnomedicinal plants

Species	Family			Tropical			Sub-tropical			Temperate		
**Trees (ha**^**-1**^**)**		**Ethnomedicinal uses**	**Part used**	**Density**	**TBC**	**IVI**	**Density**	**TBC**	**IVI**	**Density**	**TBC**	**IVI**

*Acacia catechu *(L.f.) Willd.	Fabaceae	digestive purposes, respiratory diseases, diarrhea, dysentery, bronchitis, menstrual disorder	W,B	88	2.08	29.22	106	2.40	41.20	-	-	-

*Aegle marmelos *(L.) Corrêa	Rutaceae	digestive disorders	F	52	0.74	19.19	88	1.34	34.08	-	-	-

*Cassia fistula *L.	Fabaceae	antiseptic, asthma, respiratory disorder	F,B	56	1.068	17.39	-	-	-	-	-	-

*Holarrhena antidysenterica *(L.) Wall. ex A. DC.	Apocynaceae	Dysentery, febrifuge	B,L,S	72	1.33	23.63	-	-	-	-	-	-

*Lyonia ovalifolia *(Wall.) Drude	Ericaceae	Wounds, boils	S	-	-	-	-	-	-	153	3.55	54.97

*Ougeinia oojeinensis *Hochr.	Fabaceae	digestive troubles	G	32	0.40	14.69	56	1.24	22.6	-	-	-

*Phyllanthus embelica *L.	Euphorbiaceae	Source of vitamin C	F	-	-	-	44	0.81	19.02	-	-	-

*Prunus cerasoides *Buch.-Ham. ex D. Don	Rosaceae	Swellings, contusions	B	-	-	-	-	-	-	84	1.73	33.76

*Pyrus pashia *Buch.-Ham. ex D. Don	Rosaceae	digestive disorders	F	-	-	-	-	-	-	82	1.75	30.87

*Quercus leucotrichophora *A. Camus	Fagaceae	gonorrheal and digestive disorders	G	-	-	-	-	-	-	219	5.02	71.14

*Rhododendron arboreum *Sm.	Ericaceae	digestive and respiratory disorders	F,B	-	-	-	-	-	-	160	4.40	62.19

*Terminalia belerica *Roxb.	Combretaceae	Fruit is ingredient of Trifala	F	32	1.28	20.34	32	0.74	11.43	-	-	-

*Terminalia chebula *Retz.	Combretaceae	Fruit is ingredient of Trifala	F	-	-	-	32	1.34	14.19	-	-	-

*Terminalia tomentosa *(Roxb.) Wight &Arn.	Combretaceae	liver troubles	B	24	0.57	15.09	36	1.34	17.34	-	-	-

**Shrubs (ha**^**-1**^**)**												

*Adhatoda vasica *Nees in Wallich, Pl. Asiat. Rar.	Acanthaceae	cough, cold, pulmonary affections, bronchitis and fever	F,L,T	364	0.041	60.79	394	0.062	36.45	-	-	-

*Berberis asiatica *Roxb.	Berberidaceae	ophthalmic	R	-	-	-	-	-	-	275	0.034	77.80

*Calotropis procera *(Aiton). W.T. Aiton	Asclepiadaceae	expectorant, cough, cold, asthma	R,F	92	0.007	16.96	-	-	-	-	-	-

*Colebrookea oppositifolia *Sm.	Lamiaceae	wounds	L	72	0.008	13.48	-	-	-	-	-	-

*Cotoneaster bacillaris *Wall. Kurz ex Lindl.	Rosaceae	scabies and rheumatic arthritis	L	-	-	-	-	-	-	72	0.009	26.83

*Indigofera gerardiana *Wall. ex Baker	Fabaceae	diarrhea, dysentery and cough.	L				252	0.063	29.25	-	-	-

*Leptodermis lanceolata *Wall.	Rubiaceae	migraines	B	-	-	-	-	-	-	116	0.011	28.79

*Prinsipia utilis *Royle	Rosaceae	rheumatic pains, diarrhea	S,B	-	-	-	-	-	-	180	0.042	41.86

*Rhus parviflora *Roxb.	Anacardiaceae	Cholera	L				284	0.113	36.22	88	0.015	26.49

*Woodfordia fructicosa *L.	Lythraceae	febrifuge	L				316	0.092	37.33	-	-	-

**Herbs (m**^**2**^**)**												

*Achyranthes aspera *L	Amaranthaceae	malarial fever, delivery, dropsy, bronchitis	WP	0.36	0.025	15.33	0.08	0.009	9.08	-	-	-

*Aerva sanguinolenta *(L.) Blume	Amaranthaceae	Diuretic, demulcent.	WP	0.37	0.06	25.68	0.12	0.011	9.08	-	-	-

*Ageratum conyzoides *L.	Asteraceae	sores, cuts, skin ailments	WP	-	-	-	0.15	0.016	10.11	-	-	-

*Ajuga brachystemon *Maxim.	Lamiaceae	febrifuge	L	0.17	0.009	9.88	-	-	-	-	-	-

*Anagallis arvensis *L.	Primulaceae	leprosy, dropsy,cerebral affections	WP	-	-	-	-	-	-	0.26	0.004	9.19

*Barleria prionitis *L.	Acanthaceae	Cough, cold	R,B	0.21	0.004	8.86	0.20	0.006	11.44	-	-	-

*Bidens bipinnata *L.	Asteraceae	Leprosy, cures	L	0.17	0.023	11.91	-	-	-	-	-	-

*Boerhavia diffusa *L.	Nyctaginaceae	Asthma, bronchitis, energy tonic	WP	0.32	0.007	11.86	0.26	0.007	13.35	-	-	-

*Commelina benghalensis *L.	Commelinaceae	Dysentery, swelling, ache.	WP	0.21	0.026	13.12	0.19	0.001	13.51	-	-	-

*Cynodon dactylon *(L.) Pers.	Poaceae	fever	R	0.42	0.002	11.49	0.25	0.032	21.42	-	-	-

*Cynoglossum glochidiatum *Wall. ex Benth.	Boraginaceae	Dyspepsia, digestive.	R	0.19	0.024	13.39	0.28	0.032	21.87	-	-	-

*Desmodium elegans *DC.	Fabaceae	carminatives	R	0.20	0.23	11.38	-	-	-	-	-	-

*Euphorbia hirta *L.	Euphorbiaceae	bronchial infection, asthma, warts	WP	-	-	-	0.22	0.006	11.96	-	-	-

*Geranium ocellatum *Cambess.	Geraniaceae	liver troubles, fever	WP	-	-	-	-	-	-	0.35	0.033	18.76

*Imperata cylindrica *L.	Poaceae	tonic	R	-	-	-	-	-	-	0.33	0.004	9.18

*Launaea asplenifolia *Hook. f.	Asteraceae	diarrhea	R	-	-	-	-	-	-	0.35	0.003	11.94

*Leucus indica *(L.) R. Br. Ex Vatke	Lamiaceae	Wound, sores	L	0.16	0.026	11.05	-	-	-	-	-	-

*Mentha arvensis *L.	Lamiaceae	Vomiting, indigestion	WP	-	-	-	-	-	-	0.25	0.031	14.92

*Micromeria biflora *(Buch.-Ham. ex D. Don) Benth.	Lamiaceae	gastroenteritis	L	-	-	-	0.45	0.015	17.32	-	-	-

*Origanum vulgare*	Lamiaceae	bronchitis, colic, diarrhea	WP	0.27	0.07	24.74	-	-	-	-	-	-

*Oxalis corniculata *(DC.) Raeusch	Oxalidaceae	Cataract, conjunctivitis	L	0.33	0.002	11.49	0.025	0.032	21.42	-	-	-

*Phyllanthus amarus *Schumach. & Thonn.	Euphorbiaceae	astringent, stomachic, diuretic, febrifuge	WP	0.42	0.002	14.07	0.22	0.003	10.24	-	-	-

*Pimpinella diversifolia *DC.	Apiaceae	Cough, cold, digestive disorders	WP	-	-	-	0.37	0.004	15.13	-	-	-

*Ranunculus sceleratus *L.	Ranunculaceae	Vermifuge, skin disorders	WP	0.18	0.004	10.53	-	-	-	-	-	-

*Roylae cinerea *(D.Don) Baill.	Lamiaceae	malarial fever	L	0.17	0.014	9.13	-	-	-	-	-	-

*Rumex hastatus *D. Don	Polygonaceae	Cuts, wounds,check bleeding	L	0.20	0.03	13.80	0.10	0.005	11.49	-	-	-

*Saponaria vaccaria *L.	Caryophyllaceae	bile complaints	WP	-	-	-	-	-	-	0.27	0.005	12.80

*Sida acuta *Burm.f	Malvaceae	Demulcent, diuretic, leucorrhoea	L, R	0.34	0.054	21.06	-	-	-	-	-	-

*Sida cordifolia *L.	Malvaceae	dyspepsia, astringent, diuretic	S,R	0.44	0.101	32.31	0.155	0.004	39.54	-	-	-

*Swertia angustifolia *Buch.-Ham. ex D.Don	Gentianaceae	febrifuge	WP	-	-	-	-	-	-	0.23	0.023	14.92

*Tridax procumbens *L.	Asteraceae	Wounds, cuts	WP	0.39	0.002	13.37	0.34	0.043	27.35	-	-	-

*Vernonia anthelmintica *(L) Willd.	Asteraceae	intestinal disorders, fever, skin ailments	WP	-	-	-	-	-	-	0.26	0.053	21.84

*Potentilla gerardiana *Lindey ex Lehmann	Rosaceae	wounds	R	-	-	-	-	-	-	0.23	0.05	14.9

W = wood; B = Bark; F = Fruit; L = Leaf; S = Seed; G = Gum; F = Flower; T = Twigs; R = Root; WP = Whole Plant

### Ethnomedicinal inventory

Information on plants with ethnomedicinal uses was collected from informants living in villages adjacent to the surrounding forest. After establishing oral prior informed consent in village meetings, about 10% of the inhabitants were interviewed about their dependence on the forest for various products, especially for medicinal purposes. The informants were randomly selected and included older men and women, well versed in the identification of plants, who regularly used and visited the forests since their childhood and used plants to cure various ailments. In the initial selection of informants younger participants were considered, but were later excluded because initial interviews indicated that they did not have much knowledge about medicinal plant use. The interviews were conducted in the local dialect to avoid translation problems. During the interviews structured questionnaires were used to obtain information on medicinal plants, including the local name of the plant, name of the disease for which a particular plant was used, part of the plant used etc. The informants were asked to show the plants in their natural habitat. Specimens of all plants were then collected and identified at the Garhwal University Herbarium (GUH), using [23].

### Consensus survey

A consensus survey was conducted based on peoples opinion on the number of plants used for a particular ailment. The consensus factor (Fic) was used to test the homogeneity of the informant's knowledge according methods described by Trotter and Logan [24] and Ragupathy et al. [25]

The consensus factor was calculated as follows

$$\text{Fic}={\text{N}}_{\text{ur}}-{\text{N}}_{\text{t}}∕\left({\text{N}}_{\text{ur}}-\text{1}\right)$$

The resulting factor ranges between 0 to 1, where a high value indicates for a high rate of informant consensus. N_ur _is the number of use-reports of informant's for a particular illness, where a use-report is a single record of the use of a plant mentioned by an individual, and N_t _refers to the number of taxa (species) used for a particular illness category by all informants.

## Results and Discussion

### Ethnomedicinal uses

Ethnobotany is not new to India [26] with over 400 different tribal and other ethnic groups [27,28]. Ethnobotanical information on medicinal plants and their uses by indigenous cultures is useful not only for the conservation of traditional knowledge and biodiversity, but also to promote community health care, and might serve in drug development. The information can provide a guide for drug development, assuming that a plant that has been used by indigenous people over a long period of time may well have an allopathic application [29,30].

**Figure 2:**
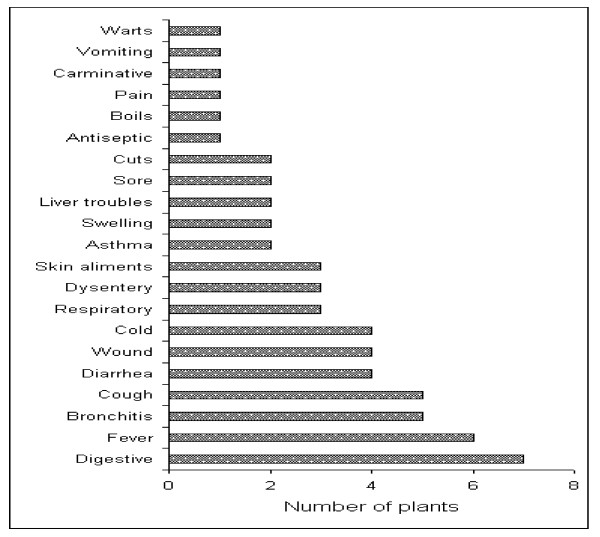
**Number of plants used for different disease curing**.

Table 2 provides the scientific names for all plants collected, as well as information on the parts used. Overall 14 trees, 10 shrubs, and 33 herbs were identified. These plants were used to treat a total of 47 diseases, ranging from simple to highly complicated, including asthma and respiratory problems. The greatest number of plants (7) was used for digestive disorders, followed by fever (6) and bronchitis (5). A single species was recorded to treat each of the following ailments: warts, vomiting, carminative, pains, boils, and much other species (Figure 2).

A comparative study in Bhotiya tribal communities in the Central Himalaya found that eighty-six plant species were identified as being used for treatment of 37 common ailments [31].

A study on the status of medicinal plants in Uttarakhand Himalaya [32] found a total of 243 medical herbal formulations prepared by Vaidyas (healers) treating 73 different ailments. Plants were used as the major ingredients for these medical formulations. A total of 156 medicinal plant species were documented during the survey. Of these 55% were cultivated and 45% were wild collected.

The plants found in the present study are distributed among 30 plant families. The largest number of species (7) belonged to the Lamiaceae followed by Asteraceae, Rosaceae, and Fabaceae with five species each (Table 3). A study of medicinal plants in the trans-Himalayan arid zone of Mustang district, Nepal, also found the largest numbers of medicinal plants belonged to the Lamiaceae [33].

**Table 3:** Distribution of herbs, shrubs and trees in different families

Family	Herb	Shrub	Tree	Total
Acanthaceae	1	1	-	2
Amaranthaceae	2	-	-	2
Apiaceae	1	-	-	1
Asteraceae	5	-	-	5
Boraginaceae	1	-	-	1
Caryophyllaceae	1	-	-	1
Commelinaceae	1	-	-	1
Euphorbiaceae	2	-	-	2
Rosaceae	1	2	2	5
Fabaceae	1	1	3	5
Gentianaceae	2	-	-	2
Lamiaceae	6	1	-	7
Malvaceae	2	-	-	2
Nyctaginaceae	1	-	-	1
Oxalidaceae	1	-	-	1
Poaceae	2	-	-	2
Polygonaceae	1	-	-	1
Ranunculaceae	1	-	-	1
Primulaceae	1	-	-	1
Rutaceae	-	-	1	1
Apocynaceae	-	-	1	1
Ericaceae	-	-	2	2
Euphorbiaceae	-	-	1	1
Fagaceae	-	-	1	1
Combretaceae	-	-	3	3
Berberidaceae	-	1	-	1
Asclepiadaceae	-	1	-	1
Rubiaceae	-	1	-	1
Anacardiaceae	-	1	-	1
Lythraceae	-	1	-	1

Total	33	10	14	57

A field survey conducted in four different places of Kathmandu valley recorded thirty six medicinal plants used to treat ailments such as diarrhoea, stomach ache, gastritis, jaundice, bodyache, bleeding, etc. [34]. The results indicate that inhabitants of the Kathmandu valley still rely on traditional medicines for their primary health care. The indigenous knowledge of local traditional healers in the Kancheepuram district of Tamilnadu also showed that many people still continue to depend on medicinal plants at least for the treatment of common health problems [35].

**Figure 3:**
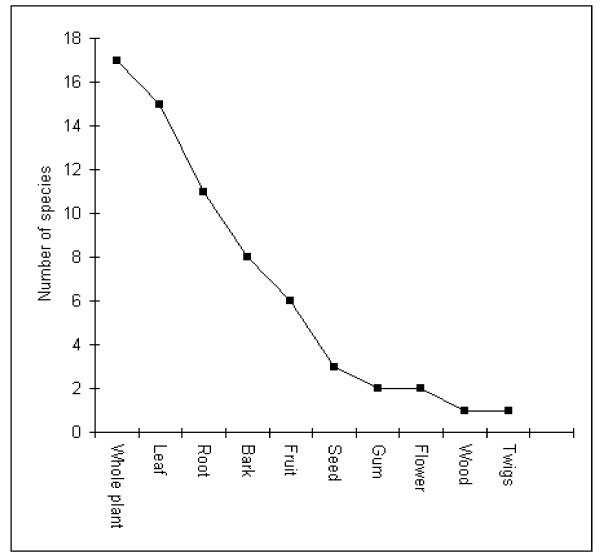
**Number of plants parts used for disease curing**.

In the present study most preparations used the whole plant, followed by leaf and roots (Figure 3). A study conducted in Chakrata Forest Division, Uttarakhand, showed a higher incidence of root, leaf, and bark use to treat various diseases [36].

Traditional medicines are a central component in health care systems in developing countries, where up to 80% of the population depends on traditional medical systems. The use of herbal medicines, is also increasing in developed countries, based on the belief that herbal remedies are safe because of their natural origin [37]. Globally, there are about 120 plant-derived drugs in professional use; three quarters of which are obtained from traditional medicinal plants [38]. Unfortunately, according to a recent report, almost one third of medicinal plant species could become extinct, with significant losses reported in China, India, Kenya, Nepal, Tanzania, and Uganda [39]. Greater losses are expected to occur in arid and semi-arid areas due to the impact of climate change, erosion, expansion of agricultural land, wood consumption, and exploitation of natural vegetation, increased global trade in natural resources, domestication, selection and grazing [40].

The traditional plant knowledge however is disappearing in many communities because of rapid socioeconomic and cultural change. The sustained use of this knowledge and its documentation is therefore essential.

### Ecological status

In the present study, the distribution of species ranged from altitude 350 m to 2300 m m.a.s.l. The vegetation composition varied with changes in altitude. Plant resources distributed across complex landscapes modified for human needs [11]. For example, conservation agencies recognize geographically uneven occurrences of high species richness and rare and endemic plants in their designation of biodiversity hotspots [40-42] or ecoregions [43,44]. These localities are then prioritized by the degree to which human activities threaten existing patterns [45]. Diversity patterns at different geographic scales, however, may be created or degraded by physical-environmental conditions and human-historical processes that influence resource availability and habitat heterogeneity [46,47].

In this study, we focused on the ecology and ethnomedicine of woody and herbaceous plants. These plants are distributed in highly-fragmented habitats, and are potentially threatened. Among the high peaks of the Himalaya, local inhabitants were found to inflict a great deal of pressure on medicinal plant populations because at higher altitudes health care facilities were almost nonexistent, and people met their medicinal requirements with forest products. At lower altitudes, people also used medicinal plants, but owing to better infrastructure, they also used nearby health centers for the treatment various diseases.

To promote a conservation agenda, it is important to understand how local communities use and manage natural resources. Studies in ethnobiology (including ethnobotany) and traditional ecological knowledge are known to serve as significant bridges between conservation scientists and local communities. These studies help to explain how local communities relate to their environment and hence, suggest ways to promote their active involvement in natural resource conservation [48].

The ecological information of plants is given in Table 2. A total of 57 species were recorded from all three regions (tropical, sub-tropical and temperate). Among the trees, *Acacia catechu*, *Aegal marmelose*, *Ougeinia oojeinensis*, *Terminalia belerica*, and *Terminalia tomentosa *were common in the tropical and sub-tropical regions. *Acacia catechu *was dominant in the tropical and sub-tropical regions. *Ougeinia oojeinensis *was the least common tree in the tropical region and *Terminalia belerica *in sub-tropical region. In the temperate region, *Quercus leucotrichophora *was dominant and *Pyrus pashia *least dominant. Other associated species are shown in Table 2. In the shrub layer, a total of 10 species were recorded from all regions: Three species were found in tropical areas, 4 in sub-tropical areas, and 5 in temperate areas. *Adhatoda vasica *was found in both tropical and sub-tropical regions, while *Rhus parviflora *occurred in both sub-tropical and temperate regions. Among the 33 species of herbs, *Sida cordifolia *was dominant in tropical and sub-tropical region while least dominant herb in the tropical region was *Barleria prionitis*, and in the sub-tropical region *Achyranthes aspera *and *Aerva sanguinolenta*. In the temperate areas, the dominant and least dominant species were *Vernonia anthelmintica *and *Imperata cylindrical *respectively. Other associated herbs are given in Table 2.

**Figure 4:**
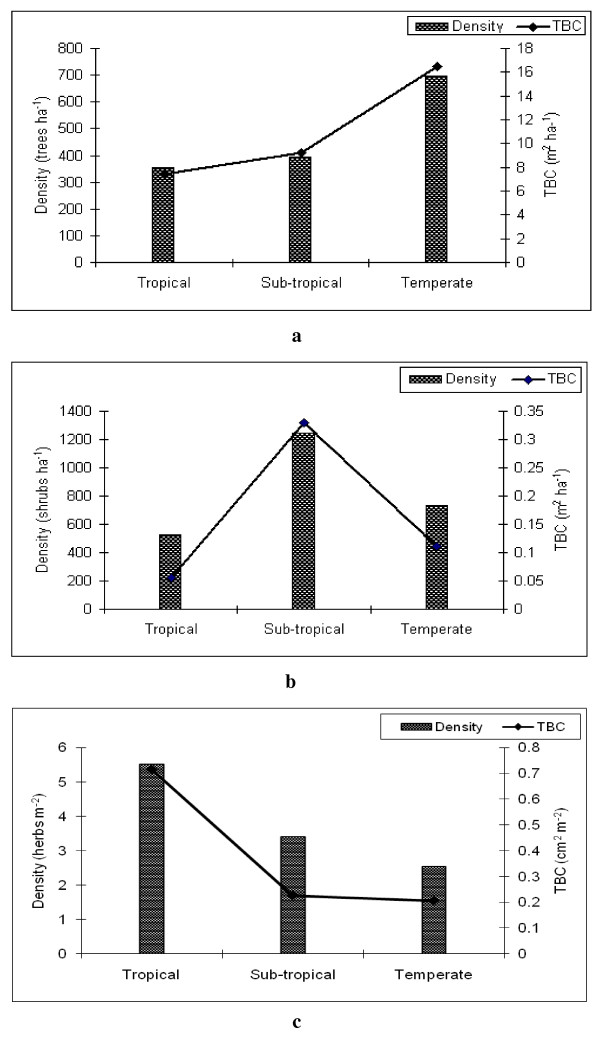
**Total density and total basal cover of various areas at each altitude**. Trees (4a); shrubs. (4b) and herbs (4c).

The density and total basal cover of trees, shrubs and herbs in the tropical, sub-tropical and temperate regions is shown in Figures 4a, 4b, and 4c. In the tree layer the highest value of density (698 trees ha^-1^) and total basal cover (16.45 m^2 ^ha^-1^) was in temperate region followed by sub-tropical and tropical regions (Figure 4a). In the shrub layer the density and total basal cover was highest in sub-tropical region and lowest in the tropical region (Figure 4b). In the herb layer the trend of density and total basal cover was inverse to the tree layer and highest in the tropical region, followed by the sub-tropical and temperate regions (Figure 4c). The trend of tree density and total basal cover increased with increasing altitudes. Shrubs were increasing in the sub-tropical region. The dependency of the villagers on medicinal plants increased with increasing altitudes due to increasing lack in healthcare facilities. Herb density and total basal cover were reduced with altitude, which could be the main effect of exploitation of these medicinal herbs for human health.

Most of the informants suggested that medicinal plants are an important source for daily healthcare and the associated knowledge was traditionally transmitted. They also suggested that these species help maintain the ecological balance of the area by decreasing soil erosion and increasing moisture in the soil, thus improving conditions for human and livestock needs. Most of the respondent said however that they did not apply any management or paid any attention to conservation needs of the species because of lack ecological knowledge. People were well aware that deforestation, overgrazing, and overexploitation of the species in a particular region may lead to the extinction of this valuable resource. The changing ecological situation was recognized as a main reason for severe problems like forest fires, erosion and drought, as well as for the disappearance of important medicinal species. The expansion of agriculture, and logging was mentioned as clearly reducing the population of highly valuable medicinal plants.

### Consensus survey of medicinal plants

The consensus survey indicated that six plant species were used most commonly for individual diseases, and therefore the informant's consensus index factor was high (1.0). Two taxa were often used for five other diseases (the index factor range was 0.75 to 0.98). Digestive diseases were cured with the highest number of taxa (7) and its consensus index factor was 0.84 (Table 4). The local population had a very high level of agreement on the usages of plants for specific ailments. The index value was high for warts, vomiting, carminative, pain, boils and antiseptic uses (1.0), and lowest for bronchitis (0.33) were five (5). Ragupathy et al. [25] published first consensus analysis research for aboriginal group in India. The consensus report of northeast India is also carried out by Sajem and Gosai [49].

**Table 4:** Consensus index for ethnomedicinal plants

Illness category	**Number taxa used (N**_**t**_**)**	**Number of use-reports (N**_**ur**_**)**	**Informant's consensus index factor (F**_**ic**_**)**^**a**^
Warts	1	22	1.0
Vomiting	1	29	1.0
Carminative	1	15	1.0
Pain	1	40	1.0
Boils	1	21	1.0
Antiseptic	1	39	1.0
Cuts	2	51	0.98
Sores	2	39	0.92
Liver troubles	2	5	0.75
Swelling	2	61	0.98
Asthma	2	15	0.93
Skin aliments	3	25	0.92
Dysentery	3	31	0.93
Respiratory	3	10	0.78
Cold	4	13	0.75
Wound	4	42	0.93
Diarrhea	4	16	0.80
Cough	5	13	0.67
Bronchitis	5	7	0.33
Fever	6	16	0.67
Digestive	7	39	0.84

^**a**^F_ic _= N_ur_-N_t_/(N_ur_-1), providing a value between 0 and 1, where high value indicates a high rate of informant consensus.

## Conclusions

The results of this study indicate that medicinal plants are used frequently by local people in the region. Some of the plants are already under threat because of overexploitation, including clearing land for agriculture, encroachment and abrupt change in environmental conditions. The majority bulk of the inhabitants seem to be unaware of the great threat to medicinal plants growing in the wild. The data presented here helps to fill this educational and awareness gap. In particular, the importance of these medicinal plants for treating various diseases must be emphasized, and their preservation and sustainable use must be included in future conservation strategies.

## Competing interests

The authors declare that they have no competing interests.

## Authors' contributions

MK designed the study, preformed the field survey, and prepared the draft manuscript, MAS analyzed the data and prepared the draft manuscript, RWB revised the manuscript and data analysis. All authors read and approved the final manuscript.
